# Level of blood pressure control among hypertensive patients on follow-up in a Regional Referral Hospital in Central Kenya

**DOI:** 10.11604/pamj.2014.18.278.4308

**Published:** 2014-08-05

**Authors:** Ernest Muthami Mutua, Moses Mwangi Gitonga, Beth Mbuthia, Nelly Muiruri, Joyce Jebet Cheptum, Thomas Maingi

**Affiliations:** 1 School of Health Sciences, Dedan Kimathi University of Technology, Nyeri, Kenya; 2 Nyeri Provincial General Hospital, Nyeri, Kenya; 3 School of Health Sciences, Kenyatta University, Nairobi, Kenya

**Keywords:** Hypertension, blood pressure, diabetes, antihypertensive, Kenya

## Abstract

**Introduction:**

Uncontrolled hypertension is a leading modifiable risk factor for cardiovascular disease morbidity and mortality. Data on adequacy of blood pressure control in Kenya is scarce. This study aimed at assessing the level of blood pressure control among hypertensive patients on follow-up in a regional referral hospital.

**Methods:**

Data regarding blood pressure, antihypertensive medication use, and comorbidities was abstracted from medical records of 452 hypertensive patients seen in Nyeri Provincial General Hospital between January and March 2013. Adequate blood pressure control was defined as a systolic pressure < 140 mmHg (< 130 mmHg for diabetic hypertensive patients) and a diastolic pressure < 90 mmHg (< 80 mmHg for diabetic hypertensive patients). Data was entered and analyzed using STATA 9 (StataCorp, Inc, Texas, USA).

**Results:**

Only 33.4% of patients had a blood pressure within the recommended limits. In multivariate analysis, using a calcium channel blocker was significantly associated with good blood pressure control (OR, 2.1; 95% CI, 1.4, 3.3). On the other hand, old age (≥ 60 years), being diabetic, and the use of three or more antihypertensive drugs were associated with reduced odds of good blood pressure control (OR, 0.64; 95% CI, 0.43; OR, 0.54; 95% CI, 0.36, 0.81; and OR, 0.41; 95% CI, 0.26, 0.64, respectively).

**Conclusion:**

Poorly controlled blood pressure is an important public health concern among hypertensive patients in this region. Elderly patients, those with diabetes, and those on multidrug regimens are at higher risk for poor blood pressure control and warrant closer attention.

## Introduction

Hypertension is an important public health concern worldwide. According to the World Health Organization (WHO), the global prevalence of hypertension among adults aged 25 years and over was 40% in 2008 [1]. The total number of people with high blood pressure worldwide grew from 600 million in 1980 to almost 1 billion in 2008 [1]. Compared with other parts of the world, Africa bears the highest burden of hypertension, with an overall prevalence of 40% [1]. High blood pressure is a major problem in the health care system because of its association with an increased risk of cardiovascular disease; the risk for stroke and coronary heart disease increases progressively as blood pressure increases [2]. Other complications associated with hypertension include heart failure, peripheral vascular disease, renal impairment, retinal hemorrhage, and visual impairment [3].

Lowering blood pressure in hypertensive patients reduces the risk for cardiovascular events [4, 5]. Thus, ongoing surveillance to determine trends in blood pressure is necessary to provide a basis for evaluating and guiding public health interventions. However, there is little data on blood pressure control among people with hypertension in Kenya; therefore, it is difficult to characterize the national burden. Currently, only three studies have been performed and the results suggest a wide range of blood pressures. One community study performed in Nakuru, in the Rift Valley region of Kenya, found that only 29% of patients receiving drugs actually had their blood pressure within the recommended levels (< 140/90 mmHg) [6]. Another study performed in Nandi district reported a much lower level of blood pressure control, with only 2.6% of patients on drug treatment having a blood pressure under control [7]. The other study done in Kenyatta National Hospital, the country's premier referral hospital, indicated that only 26% of hypertensive patients on follow-up in the hospital's medical outpatient clinics had well-controlled blood pressure [8].

Despite the fact that central Kenya accounts for the highest proportion of hypertension-related outpatient clinic attendances [9], there is no data to show the extent to which patients on follow-up have their blood pressure under control. Therefore, the aim of this study was to determine the level of blood pressure control and characterize antihypertensive medication use and comorbidity profiles among hypertensive patients being followed-up at Nyeri Provincial General Hospital, central Kenya.

## Methods

### Description of the survey and study population

The study was a cross sectional survey including 452 hypertensive patients who were seen at Nyeri Provincial General Hospital between January and March 2013. Nyeri hospital is a referral health-care facility serving a wide region within central Kenya (population of 4,383,743 persons as of 2009 national census) [10]. The hospital runs a busy medical outpatient clinic (MOPC) on Tuesdays and a diabetic outpatient clinic (DOPC) on Fridays. Non-diabetic hypertension patients are usually seen in the MOPC, while those with diabetes are seen in the DOPC.

Data was collected from patient files retrieved from the hospital's medical records department. The patient files were selected by systematic random sampling from the list of hypertension patients seen on each clinic day in both the MOPC and DOPC. The sample size was calculated using the formula for proportion (Sample size **n**=**(Np(1-p)/(d^2^/Z^2^_1-α/2_)((N-1)+p)(1-p)**; the *Open Source Epidemiologic Statistics for Public Health, Version 2.3.1* was applied in the calculations [11]. At 99% confidence level, a total of 464 hypertensive patients would be required to estimate 26% of hypertensive patients with adequate blood pressure control; this computation was corrected for an estimated population size of 5000 patients. For the 24 clinics held during the study period, a minimum of 19 patients were required per clinic in order to meet the study's sample size. Since it was anticipated that patient files may not be available for all the clinics, the recruitment target was raised to 25 patients per clinic. The sampling interval was determined by dividing the total number of hypertensive patients seen on each clinic day by 25. A number between one and the sampling interval was chosen using random number tables; this was the index case. A sticker was placed on each of the selected files to avoid resampling at subsequent clinics. Newly diagnosed patients were excluded from the study.

A data abstraction form was used to collect information about the patients’ systolic blood pressure (SBP) and diastolic blood pressure (DBP) readings recorded at their clinic visits. Other data included demographics (age, gender), current antihypertensive medication, and comorbidities. A trained medical records clerk performed the retrieval of patient files and data abstraction. The study underwent ethical review and clearance by the Kenya National Council for Science and Technology. Permission to access the patients’ medical records was sought from the management board of Nyeri Provincial General Hospital. All patient information was treated with utmost confidentiality, and no personal identifiers were included in the data abstraction forms.

### Data analysis

All data were analyzed using STATA 9 (StataCorp, Inc, Texas, USA). The percentage of patients with well controlled blood pressure was determined according to guidelines provided in the Seventh Report of the Joint National Committee on Prevention, Detection, Evaluation, and Treatment of High Blood Pressure (JNC7) [12]. For non-diabetic hypertensive patients, good control was defined as BP < 140/90 mmHg, while that for diabetic hypertensive patients was < 130/80 mmHg. All patients with BP ≥ 140/90 mmHg were further sub-categorized into stage 1 (SBP 140-159 mmHg or DBP 90-99 mmHg) and stage 2 (SBP ≥ 160 mmHg or DBP ≥ 100 mmHg). The prevalence of isolated uncontrolled systolic and diastolic hypertension was also established by determining the number of patients with SBP ≥ 140 mmHg and DBP < 90 mmHg, and DBP ≥ 90 mmHg and SBP< 140 mmHg, respectively.

To identify covariates significantly related to blood pressure control, a combined analysis of all patients was conducted, with good blood pressure control being defined as SBP < 140 mmHg and DBP < 90 mmHg. Firstly, a bivariate analysis (χ^2^ test) was performed to examine the relationship between blood pressure control and sex, age, number of drugs in the antihypertensive regimen, class of medication (diuretics, calcium channel blockers (CCBs), angiotensin converting enzyme inhibitors (ACEIs), angiotensin receptor blockers (ARBs) and beta blockers), and comorbidities. Secondly, a multiple binary logistic regression model was fitted using a forward selection approach, with variables significant at P < 0.05 being added to identify predictors of blood pressure control.

## Results

### Sample characteristics and comorbidity profile

The sample comprised 452 hypertensive patients (72.6% female). The mean age was 63 (+/- 12.4) years. Non-diabetic hypertensive patients accounted for 58.2% (263) of the study sample, with the remaining 41.8% (189) being hypertensive patients with diabetes. The other comorbid conditions documented in the patients’ medical records were as follows: cardiovascular disease (4%); asthma (3.3%); peptic ulcer disease (1.1%); hyperthyroidism (1.3%); and others (6.9%). Among the patients with cardiovascular disease (18/452), heart failure was the commonest comorbidity (2%), followed by stroke (1.3%) and ischemic heart disease (0.7%). Renal disease was not present in any of the patients reviewed.

### Blood pressure control

Of the 263 non-diabetic hypertensive patients, only 128 (48.7%) had a blood pressure < 140/90 mmHg. Among the diabetic hypertensive patients (n=189), only 23 (12.1%) had a blood pressure < 130/80 mmHg. Hence, the percentage of patients with good blood pressure control was 33.4% (151/452). In the combined analysis, involving both non diabetic and diabetic hypertensive patients (N=452), only 196 (43.4%) of the study subjects had a blood pressure < 140/90 mmHg. Of the remaining 256 patients with uncontrolled hypertension, 62.5% were at stage 1 and 37.5% were at stage 2. The prevalence of isolated uncontrolled SBP was more than twice that of isolated uncontrolled DBP (29.7% vs. 11.7%). Regarding antihypertensive medication use, 82.1% (161/196) required two or more drugs to have their blood pressure controlled. Diuretics were the most commonly prescribed drugs (78.1%), with the majority being thiazides. The antihypertensive medications prescribed are listed in Table 1.

**Table 1:** Antihypertensive medication use by patients in the study

Drug	n (%)
**Diuretics**	
Thiazides	328 (72.6)
Furosemide	15 (3.3)
Aldactone	10 (2.1)
**CCBs**	
Nifedipine	129 (28.5)
Amlodipine	39 (8.6)
**Beta blockers**	
Atenolol	139 (30.8)
Propranolol	8 (1.8)
Carvedilol	16 (3.5)
**ACEIs**	
Enalapril	241(53.3)
**ARBs**	
Lorsatan	98 (21.7)
**Number of prescribed antihypertensive drugs**	
1	75 (16.6)
2	191 (42.3)
≥ 3	186 (41.2)

ACEIs: Angiotensin converting enzyme inhibitors; ARBs: Angiotensin receptor blockers; CCBs: Calcium channel blockers. Total sample (N): 452

### Predictors of blood pressure control

Bivariate analysis revealed that diabetes status and the number of prescribed antihypertensive drugs were significantly associated with BP control (χ^2^; p< 0.05). The results of bivariate analysis between blood pressure control status and the other study variables are summarized in Table 2. In multivariate analysis, being on a CCB was significantly associated with well-controlled blood pressure (OR, 2.1; 95% CI, 1.4, 3.3). However, the odds of good control were significantly reduced if the patient was aged 60 years and over (OR, 0.64; 95% CI, 0.43, 0.96), had diabetes (OR, 0.54; 95% CI, 0.36, 0.81), or was taking three or more antihypertensive drugs (OR, 0.41; 95% CI, 0.26, 0.64). The results of the multiple logistic regression analysis conducted are presented in Table 3.

**Table 2:** Bivariate analysis of the association between blood pressure control and other study variables

Variable	Total	Blood Pressure		Test	P value
		Controlled n (%)	Uncontrolled n (%)		
**Sex**				≡ ^2^	0.31
Male	124	49 (39.5)	75 (60.5)		
Female	328	147 (44.8)	181(55.2)		
**Age**				≡ ^2^	0.054
< 60 years	162	80 (49.4)	82 (50.6)		
≥ 60 years	290	116 (40)	174 (60)		
**Number of prescribed antihypertensive drugs**				≡ ^2^	0.008
≤ 2	266	129 (48.5)	137 (51.5)		
≥ 3	186	67 (36)	119 (64)		
**On a diuretic**				≡ ^2^	0.367
Yes	353	157 (44.5)	196 (55.5)		
No	99	39 (39.4)	60 (60.1)		
**On a CCB**				≡ ^2^	0.046
Yes	168	83 (49.4)	85 (50.6)		
No	284	113 (39.8)	171 (60.2)		
**On a beta blocker**				≡ ^2^	0.056
Yes	163	61 (37.4)	102 (62.6)		
No	289	135 (46.7)	154 (53.3)		
**On an ACEI**				≡ ^2^	0.046
Yes	241	94 (39)	147 (61)		
No	211	102 (48.3)	109 (51.7)		
**On an ARB**				≡ ^2^	0.206
Yes	98	37 (37.8)	61 (62.2)		
No	354	159 (44.9)	195 (55.1)		
**Diabetic**				≡ ^2^	0.007
Yes	189	68 (36)	121 (64)		
No	263	128 (48.7)	135 (51.3)		
**Cardiovascular disease**				≡ ^2^	0.696
Present	18	7 (38.9)	11 (61.1)		
Absent	434	189 (43.6)	245 (56.5)		
**Other comorbidities**				≡ ^2^	0.459
Present	47	18 (38.3)	29 (61.7)		
Absent	405	178 (44)	227 (56)		

ACEIs: Angiotensin converting enzyme inhibitors; ARBs: Angiotensin receptor blockers; CCBs: Calcium channel blockers. Total sample (N): 452

**Table 3:** Logistic regression analysis of factors associated with good blood pressure control

Variable	OR (95% confidence interval)	P value
***On a CCB***		
No	1	
Yes	2.1 (1.4, 3.3)	0.001
***Number of prescribed antihypertensive drugs***		
≤ 2	1	
≥ 3	0.41 (0.26, 0.64)	< 0.001
***Diabetic***		
No	1	
Yes	0.54 (0.36, 0.81)	0.003
***Age***		
< 60 years	1	
≥ 60 years	0.64 (0.43, 0.96)	0.032

CCB: Calcium channel blocker

## Discussion

In the present study, we found out that only 33.4% of the hypertensive patients on follow-up at Nyeri Provincial General Hospital had their blood pressure controlled within the recommended levels. However, the level of blood pressure control reported herein is slightly better than that reported by a study performed at the country's national referral hospital (Kenyatta National Hospital) in 2009 [8], which showed that only 26% of hypertensive patients had their blood pressure under control. The reason for this discrepancy can be attributed to the fact that central Kenya (where the present study was conducted) has a high burden of hypertension [9], therefore, there is more likelihood of heightened awareness of the problem. The difference in control rates may also represent a secular trend in blood pressure control, as has been suggested elsewhere in the literature [13].

It is also worth noting that a substantial proportion of patients with uncontrolled blood pressure were at stage 2 (SBP ≥ 160 mmHg or DBP mmHg ≥ 100), indicating a higher long-term risk of cardiovascular disease [2, 14]. We noted that the levels of uncontrolled hypertension were different with respect to SBP versus DBP: the prevalence of isolated uncontrolled SBP was twice as high as that of isolated uncontrolled DBP. A similar phenomenon was reported in a study in the USA, where the percentages of patients with uncontrolled SBP and DBP were 32.7% and 82.9%, respectively [15]. This finding is significant, given the existing evidence which shows that SBP is more important than DBP in predicting cardiovascular and renal disease in the elderly [16, 17]. The benefits of systolic blood pressure reduction have also been documented in several trials [18–20].

Multivariate analysis revealed that old age, diabetes, and the use of three or more drugs were significant predictors of poor blood pressure control. Older age has been identified as an important risk factor for poor blood pressure control in a number of studies [15, 21, 22]. Compared with non-diabetic patients, those with diabetes were significantly more likely to have poorly controlled blood pressure. This finding also agrees with those of several other studies showing lower rates of well-controlled blood pressure in diabetic hypertensive patients [23–25].

For adequate blood pressure control, majority of the patients needed two or more drugs. This finding is in agreement with the existing evidence, that combination therapy is necessary for blood pressure control [26]. However, patients taking three or more drugs were 2.4 times less likely to have well-controlled blood pressure than those on two drugs or less. This may indicate that blood pressure is more difficult to control in these patients, prompting clinicians to use more aggressive treatment regimens. The association between multidrug regimens and poor blood pressure control has also been demonstrated by other studies. For example, Knight et al. (2001) studied blood pressure control among ambulatory patients and found a positive association between a multidrug regimen and poor blood pressure control. Patients taking four or more antihypertensive medications were five times more likely to have a higher blood pressure than those taking a single drug [27]. Another study by Duggirala et al. found a similar pattern; patients taking three or more drugs were 35% more likely to have poorly controlled blood pressure [28].

Being prescribed a CCB was significantly associated with good blood pressure control, and patients taking a CCB were twice as likely to have well-controlled blood pressure than those not taking a CCB. This finding is consistent with those of other studies, in which CCBs were more beneficial in achieving good blood pressure control, particularly in the elderly [29, 30].

One important limitation needs to be taken into consideration when interpreting the findings of this study. The study involved patients being followed-up at a hospital; therefore, the findings may not reflect the picture in the general population. However, given the paucity of data in this area, the study provides an important entry point to understanding the burden and factors associated with hypertension in central Kenya and in other parts of the country.

## Conclusion

This study shows that a majority of hypertensive patients in central Kenya have poorly controlled blood pressure, and identifies the need for targeted interventions to address the problem. Elderly and diabetic patients, and those on multidrug regimens, are more likely to have poorly controlled blood pressure and require closer attention. Systolic blood pressure control should be emphasized, given that it was a particular problem in this study population. The use of calcium channel blockers should be considered as they appear to provide better blood pressure control. Further research is needed to identify barriers to effective blood pressure control and examine possible interventions to improve treatment outcomes.

## References

[1] World Health Organization (2011). Global status report on noncommunicable diseases 2010: Description of the global burden of NCDs, their risk factors and determinants.

[2] Whitworth JA (2003). 2003 World Health Organization (WHO)/International Society of Hypertension (ISH) statement on management of hypertension. Journal of hypertension..

[3] Williams B, Poulter NR, Brown MJ, Davis M, McInnes GT, Potter JF (2004). British Hypertension Society guidelines for hypertension management 2004 (BHS-IV): summary. Bmj..

[4] Ogden LG, He J, Lydick E, Whelton PK (2000). Long-term absolute benefit of lowering blood pressure in hypertensive patients according to the JNC VI risk stratification. Hypertension..

[5] Neal B, MacMahon S, Chapman N (2000). Effects of ACE inhibitors, calcium antagonists, and other blood-pressure-lowering drugs: results of prospectively designed overviews of randomised trials - Blood Pressure Lowering Treatment Trialists’ Collaboration. Lancet..

[6] Mathenge W, Foster A, Kuper H (2010). Urbanization, ethnicity and cardiovascular risk in a population in transition in Nakuru, Kenya: a population-based survey. BMC public health..

[7] Hendriks ME, Wit FW, Roos MT, Brewster LM, Akande TM, de Beer IH (2012). Hypertension in sub-Saharan Africa: cross-sectional surveys in four rural and urban communities. PloS one..

[8] Acheing L, Joshi MD, Ogola EN, Karari E (2009). Adequacy of Blood Pressure Control and Level of Adherence with Antihypertensive Therapy. East African medical journal.

[9] Kenya National Bureau of Statistics (2010). Out-Patient Morbidity Statistics in Over Five Years by Province. http://www.knbs.or.ke/index.php?option=com_phocadownload&view=category&id=73:population-and-health-statistics&Itemid=1131.

[10] Kenya National Bureau of Statistics (2009). Kenya Census Report. http://www.knbs.or.ke/index.php?option=com_phocadownload&view=category&id=73:population-and-health-statistics&Itemid=1131.

[11] Dean AG, Sullivan KM, Soe MM OpenEpi: Open Source Epidemiologic Statistics for Public Health.

[12] Chobanian A, Bakris G, Black H, Cushman W, Green L, Izzo J (2003). Seventh report of the Joint National Committee on Prevention, Detection, Evaluation, and Treatment of High Blood Pressure. Hypertension..

[13] Wang TJ, Vasan RS (2005). Epidemiology of Uncontrolled Hypertension in the United States. Circulation..

[14] Chobanian AV, Bakris GL, Black HR, Cushman WC, Green LA, Izzo JL (2003). The Seventh Report of the Joint National Committee on Prevention, Detection, Evaluation, and Treatment of High Blood Pressure: the JNC 7 report. JAMA: the journal of the American Medical Association..

[15] Lloyd-Jones DM, Evans JC, Larson MG, O'Donnell CJ, Roccella EJ, Levy D (2000). Differential Control of Systolic and Diastolic Blood Pressure: Factors Associated With Lack of Blood Pressure Control in the Community. Hypertension..

[16] Kannel WB, Gordon T, Schwartz MJ (1971). Systolic versus diastolic blood pressure and risk of coronary heart disease; The Framingham study. The American journal of cardiology..

[17] Alli C, Avanzini F, Bettelli G, Colombo F, Torri V, Tognoni G (1999). The long-term prognostic significance of repeated blood pressure measurements in the elderly: SPAA (Studio sulla Pressione Arteriosa nell'Anziano) 10-year follow-up. Archives of internal medicine..

[18] SHEP Cooperative Research Group (1991). Prevention of stroke by antihypertensive drug treatment in older persons with isolated systolic hypertension - Final results of the Systolic Hypertension in the Elderly Program (SHEP). JAMA: the journal of the American Medical Association.

[19] Staessen JA, Fagard R, Thijs L, Celis H, Arabidze GG, Birkenhager WH (1997). Randomised double-blind comparison of placebo and active treatment for older patients with isolated systolic hypertension - The Systolic Hypertension in Europe (Syst-Eur) Trial Investigators. Lancet..

[20] Liu L, Wang JG, Gong L, Liu G, Staessen JA (1998). Comparison of active treatment and placebo in older Chinese patients with isolated systolic hypertension - Systolic Hypertension in China (Syst-China) Collaborative Group. Journal of hypertension.

[21] Hyman DJ, Pavlik VN (2001). Characteristics of patients with uncontrolled hypertension in the United States. The New England journal of medicine..

[22] Ornstein SM, Nietert PJ, Dickerson LM (2004). Hypertension management and control in primary care: a study of 20 practices in 14 states. Pharmacotherapy..

[23] Majernick TG, Zacker C, Madden NA, Belletti DA, Arcona S (2004). Correlates of hypertension control in a primary care setting. American journal of hypertension..

[24] McDonald MV, Pezzin LE, Peng TR, Feldman PH (2009). Understanding the complexity of hypertensive African American home care patients: challenges to intervention. Ethnicity & disease..

[25] Olomu AB, Gourineni V, Huang JL, Pandya N, Efeovbokhan N, Samaraweera J (2013). Rate and Predictors of Blood Pressure Control in a Federal Qualified Health Center in Michigan: A Huge Concern?. The Journal of Clinical Hypertension..

[26] Flack JM, Sica DA, Bakris G, Brown AL, Ferdinand KC, Grimm RH (2010). Management of high blood pressure in Blacks: an update of the International Society on Hypertension in Blacks consensus statement. Hypertension..

[27] Knight EL, Bohn RL, Wang PS, Glynn RJ, Mogun H, Avorn J (2001). Predictors of uncontrolled hypertension in ambulatory patients. Hypertension..

[28] Duggirala MK, Cuddihy RM, Cuddihy MT, Naessens JM, Cha SS, Mandrekar JN (2005). Predictors of blood pressure control in patients with diabetes and hypertension seen in primary care clinics. American journal of hypertension..

[29] Haller H (2008). Effective management of hypertension with dihydropyridine calcium channel blocker-based combination therapy in patients at high cardiovascular risk. International journal of clinical practice..

[30] Morgan TO, Anderson AI, MacInnis RJ (2001). ACE inhibitors, beta-blockers, calcium blockers, and diuretics for the control of systolic hypertension. American journal of hypertension..

